# Supplementary material for: Perturbed structural dynamics underlie inhibition and altered efflux of the multidrug resistance pump AcrB
Source: Nat Commun. 2020 Nov 4;11:5565. doi: 10.1038/s41467-020-19397-2 (PMC7642415; doi:10.1038/s41467-020-19397-2)
Supplement: Supplementary file 1 — Supplementary Information [file 41467_2020_19397_MOESM1_ESM.pdf]

Supplementary Information

Perturbed structural dynamics underlie inhibition and altered efflux of the multidrug resistance pump AcrB

Eamonn Reading[1,*†], Zainab Ahdash[1,†], Chiara Fais[2], Vito Ricci[3], Xuan Wang Kan[3], Elizabeth Grimsey[3], Jack Stone[3], Giuliano Malloci[2], Andy M. Lau[1], Heather Findlay[1], Albert Konijnenberg[4], Paula J. Booth[1], Paolo Ruggerone[2], Attilio V. Vargiu[2], Laura J. V. Piddock[3], Argyris Politis[1]*

1. Department of Chemistry, Britannia House, 7 Trinity Street, King's College London, London, SE1 1DB, UK
2. Department of Physics, University of Cagliari, Cittadella Universitaria, S.P. Monserrato-Sestu, 09042 Monserrato (CA), Italy.
3. Antimicrobials Research Group, Institute of Microbiology and Infection, College of Medical and Dental Sciences, The University of Birmingham, Birmingham, B15 2TT, UK
4. Thermo Fisher Scientific, Zwaanstraat 31 G/H, 5651 CA Eindhoven, The Netherlands

*Correspondence to: argyris.politis@kcl.ac.uk and eamonn.reading@kcl.ac.uk

†These authors contributed equally

Table of contents

Supplementary discussion of MD simulations.. ***5-9***

Supplementary figures ***10-26***

Supplementary Figure 1. AcrB central cavity and trimer architecture........................ ***10***

Supplementary Figure 2. Biophysical characterization of $AcrB^{WT}$ and $AcrB^{G288D}$ ***11***

Supplementary Figure 3. HDX-MS heat maps representing the relative fractional uptake of deuterium for peptides of AcrB ***13***

Supplementary Figure 4. HDX-MS heat maps from Supplementary Figure 3 translated onto the structure of $AcrB^{WT}$ (PDB: 2HRT)... ***14***

Supplementary Figure 5. Comparison of HDX-MS between $AcrB^{WT}$ drug-binding conditions.. ***15***

Supplementary Figure 6. Representative binding poses and RMSDs of $AcrB^{WT}$-PAβN (Pose 1 discussed in the main text) ***16***

Supplementary Figure 7. Difference in first hydration shell (Nwat) and RMSF between $AcrB^{WT}$-PAβN and apo $AcrB^{WT}$ (based on MD data from Pose 1 in Supplementary Fig. 6).. ***18***

Supplementary Figure 8. Distribution of top 200 docking poses (only the centres of mass are shown for clarity) for CIP and PAβN onto $AcrB^{WT}$ and $AcrB^{G288D}$ ***19***

Supplementary Figure 9. Representative binding poses and RMSDs of $AcrB^{WT}$-CIP-PAβN (Pose 1 discussed in the main text) ***20***

Supplementary Figure 10. Difference in first hydration shell (Nwat) and RMSF between $AcrB^{WT}$-CIP-PAβN and apo $AcrB^{WT}$ (based on MD data from Pose 1 in Supplementary Fig. 9)... ***21***

Supplementary Figure 11. Influence of drugs on $AcrB^{G288D}$ structural dynamics... .... ***22***

Supplementary Figure 12. Representative binding poses and RMSDs of $AcrB^{G288D}$-PAβN (Pose 1 discussed in the main text)................................ ***23***

Supplementary Figure 13. Difference in first hydration shell (Nwat)

and RMSF between AcrB^{G288D}-PAβN and AcrB^{WT}-PAβN

(based on MD data from Pose 1 in Supplementary Fig. 12) ***24***

Supplementary Figure 14. Representative binding poses and RMSDs

of AcrB^{G288D}-CIP-PAβN (Pose 1 discussed in the main text)..................................... ***25***

Supplementary Figure 15. Difference in first hydration shell (Nwat)

and RMSF between AcrB^{G288D}-CIP-PAβN and apo AcrB^{WT}-CIP-PAβN

(based on MD data from Pose 1 in Supplementary Fig. 14) ***26***

Supplementary Figure 16. Representative binding poses of AcrB^{WT}-CIP and AcrB^{G288D}-CIP (upper panel) and high-occupancy hydrogen bonds (H-bonds) established between CIP and the protein (lower table) ***27***

Supplementary tables ***28-36***

Supplementary Table 1. List of peptides considered in the regions of AcrB.............. ***28***

Supplementary Table 2. Number of poses, maximum and

average (pseudo) free energy of CIP and PAβN binding to AcrB^{WT} ***29***

Supplementary Table 3. Intermolecular hydrogen bonds (H-bonds)

and water-mediated interactions involving PAβN in AcrB^{WT}-PAβN

(based on MD simulations) ***30***

Supplementary Table 4. Intermolecular hydrogen bonds (H-bonds)

and water-mediated interactions involving ligands in AcrB^{WT}-CIP-PAβN

(based on MD simulations) ***31***

Supplementary Table 5. Number of poses, maximum and

average (pseudo) free energy of CIP and PAβN binding to AcrB^{G288D}....................... ***32***

Supplementary Table 6. Intermolecular hydrogen bonds (H-bonds)

and water-mediated interactions involving PAβN in AcrB^{G288D}-PAβN

(MDs data)... ***33***

Supplementary Table 7. Intermolecular hydrogen bonds (H-bonds)

and water-mediated interactions involving ligands in

$AcrB^{G288D}$-CIP-PAβN (MDs data) ***34***

Supplementary Table 8. Occupancies of intermolecular hydrogen

bonds between the ligands in $AcrB^{WT}$-CIP-PAβN and $AcrB^{G288D}$-CIP-PAβN ***35***

Supplementary Table 9. Primers used for plasmid construction

(see Methods section for more details) ***36***

Supplementary Table 10. Backbone RMSD of each pose with respect

to the X-ray crystal structure 4U95 of *E. coli* AcrB (resolution: 2.0 Å) ***37***

Supplementary Table 11. Systems considered for the analyses

of flexibility and hydration properties (based on MD simulations),

and respective reference structures ***37***

Supplementary discussion of MD simulations

In this section we report a more in-depth analysis of our MD simulations, focusing on the details of the interactions established between AcrB and the two ligands CIP and PAβN. For each system, we present the stable binding poses, which in some cases reflect ligand orientations and ligand-AcrB interactions that are representative of more than one MD replica.

AcrB^{WT}-PAβN

According to our MD simulations, an important contribution to the stabilization of PAβN in AcrB^{WT} comes from the hydrophobic trap (HT), whose residues are involved in stacking with the β-naphthylamide moiety of the inhibitor (see Pose 1 in Supplementary Fig. 6 for a representation of the binding mode). Importantly, these interactions also involve residues of the switch loop (such as F617) or belonging to adjacent regions. These findings, in agreement with previous literature[5,6], support the hypothesis that the stabilization of the switch-loop could be key to the mode of action of PAβN. This loop is also involved in the formation of stable hydrogen bonds with the amino group of the compound (see Supplementary Table 3). Additional hydrogen bonds are formed by its polar groups with polar and acid residues of the DBP, including E130, K131 (involved in interactions with the guanidino group of PAβN) and Q176 (interacting with the carbonyl group).

Important findings on the stabilization of the switch loop come from the comparison of the hydration properties of the AcrB^{WT}-PAβN complex and apo AcrB^{WT} (Supplementary Fig. 7). Indeed, despite the relevant difference in the timescales of all-atom MD simulations and typical HDX kinetics (see Methods), and while the switch loop itself moderately enhanced hydration, the nearby segments (residues 612 to 615 and 620 to 624) are overall dehydrated with respect to the *apo* form (Supplementary Fig. 7). Considerable dehydration and rigidification are also observed for part of the PBP and the CH2 entrance, consistent with HDX-MS data (Fig. 2a-b).

In agreement with previous studies[5,6], all the binding modes found for AcrB^{WT}-PAβN (Supplementary Fig. 6) feature the β-naphthylamide moiety of the inhibitor within the HT and interacting with the switch loop or nearby residues. Additional common interactions involve the aromatic rings and the amino group of the inhibitor and the PBP/DBP interface, including residues of the PN1 subdomain (such as S46, S128 and E130). Residues of this region are involved in either stacking interactions with the phenyl ring of PAβN (Supplementary Fig. 6, Pose 1, 2) or hydrogen bonds with its amino group (Pose 2; see Supplementary Table 3). Furthermore, residues belonging to segment 130-134 also interact with the guanidino group of PAβN in two representative poses (Supplementary Fig. 6, Pose 1, 3), with additional stabilization provided by Q176 and proximal residues. The other pose (Pose 2) is characterized by a different orientation of the guanidino group of the inhibitor, located in the upper part of the DBP and involved in interactions with D276 and nearby residues.

To evaluate whether the described interactions correlate with HDX-MS protection data, occupancy levels of protein-ligand hydrogen bonds and water-mediated interactions were computed (see Methods). The results (Supplementary Table 3) confirm that several residues

belonging to protected peptides in the HDX-MS (residue segments 129-137, 162-181, and 610-628) form stable direct and/or water-mediated hydrogen bonds with the inhibitor (Supplementary Table 3). Stabilization of these regions of the DBP may thus be due to the interaction with PAβN.

AcrB^{WT}-CIP-PAβN

Interactions stabilizing CIP and PAβN in AcrB^{WT} include hydrogen bonds between the two substrates (Supplementary Table 8), as well as between them and the protein (e.g. between the guanidino group of PAβN and residues E130 and D174, or between R620 and the carboxylic and carbonyl group in CIP; see Pose 1 in Supplementary Fig. 9 for a representation of the binding pose). Additional stabilization comes from stacking of aromatic rings, formed by PAβN with CIP and F615. Importantly, the direct interaction between the inhibitor and segments proximal to the switch-loop, present in AcrB^{WT}-PAβN (Supplementary Fig. 6, Pose 1), is preserved also in the presence of CIP (Supplementary Fig. 9).

The comparison of the hydration properties of AcrB^{WT}-CIP-PAβN (Supplementary Fig. 10) and AcrB^{WT}-PAβN (Supplementary Fig. 7) reveals an analogous dehydration of the residues of the binding pockets (with the exception of residues involved in interactions with the compounds, such as E173, N174 and F615 in AcrB^{WT}-CIP-PAβN and similar variations in the region surrounding the switch-loop (see Supplementary Fig. 10, Pose 1)). Such regions, involved in interactions with the substrates in both AcrB^{WT}-PAβN (Supplementary Fig. 6, Pose 1) and AcrB^{WT}-CIP-PAβN (Supplementary Fig. 9, Pose 1), is indeed considerably rigidified in both systems. This is associated with a dehydration of the segments adjacent to the loop, significantly marked in AcrB^{WT}-CIP-PAβN, and in agreement with HDX-MS data (Supplementary Fig. 10 and Fig. 2a-b).

A common trait of the binding modes found for this ternary complex is the presence of direct interactions between the two substrates, through the formation of hydrogen bonds (involving, in all poses, the carboxylic group of CIP; see Supplementary Table 8) as well as stacking of the aromatic rings (Supplementary Fig. 9, Pose 1, 3, 4). While both CIP and PAβN are located inside the DBP in three representative poses (Pose 1, 3, 4), a different binding mode is predicted with CIP located within the PBP behind the switch loop (Pose 2).

Although some differences are present, comparison of the binding regions reveals several shared traits. Firstly, interactions of at least one substrate with the HT and (the region proximal to) the switch loop are preserved. Typically, such interactions involve π-stacking with the aromatic groups of PAβN, although cation-π interactions were also observed in Pose 3 (involving e.g. F178 and the amino group of PAβN). In Pose 2 and 4, additional stacking interactions are found between CIP and the switch loop and the nearby residues.

Apart from Pose 3, another conserved trait is related to the interaction with the PBP/DBP interface. Several contacts with residues of this region (such as S46, T87, S128 and adjacent residues; see Supplementary Table 4) are formed by CIP in Poses 1 and 4, while in Pose 2 PAβN is involved in hydrogen bonds and polar interactions with E130 and nearby residues. In Pose 3, in which interactions with the PBP/DBP interface are not detected,

several contacts are formed by CIP with polar and acidic residues of the PN2 portion of the DBP, including Q151 and E152.

Comparison of such poses with 1-µs long MD simulations of $AcrB^{WT}$-CIP (see Supplementary Fig. 16 for a representative binding pose) revealed that the co-presence of PAβN determines a change in the binding region of CIP. In the absence of the EPI, indeed, CIP tends to fully occupy the HT, as reported in previous studies[29]. Although starting from different orientations than those previously reported, our MD simulations confirmed that CIP establishes strong interactions with residues F136, Y327 and F628. Moreover, according to our analyses, further stabilization comes from high-occupancy hydrogen bonds involving Q176. Stabilizing interactions do not involve residues of the PBP/DBP interface, which instead play an important role in the ternary complex, as previously mentioned.

Analysis of the occupancy of the hydrogen bonds formed by CIP and PAβN with the protein revealed that both ligands form stable interactions in all poses (Supplementary Table 4). Moreover, both ligands tend to form water-mediated interactions with the protein, frequently involving residues 128-133, 174-176 and 273-276. From the comparison with HDX-MS data, a good correlation between occupancy and protection data emerges for segments 138-149, 162-177, 263-274 (see Supplementary Fig. 10). As in the case of $AcrB^{WT}$-PAβN, therefore, intermolecular interactions formed by CIP and PAβN may be a factor for the protection of significant portions of the DBP.

$AcrB^{G288D}$-PAβN

Within $AcrB^{G288D}$-PAβN, as in the wild type protein, a significant contribution to the stabilization of PAβN comes from the residues of the HT and of the region around the switch-loop. Such residues are indeed involved in stacking with the aromatic groups of the inhibitor, as well as in cation-π interactions with its guanidino and amino groups (see Pose 1 in Supplementary Fig. 12 for a representation of the binding pose). These interactions, possibly promoted by the stable hydrogen bond formed by the amino group of PAβN with D288 (see Supplementary Table 6), were not detected in $AcrB^{WT}$-PAβN (Supplementary Fig. 6, Pose 1); thus, they provide an additional contribution to the stabilization of the inhibitor specifically for the G288D mutant. Additional contacts not observed in $AcrB^{WT}$-PAβN are formed with part of the PC1/PC2 cleft (such as L668), while interactions with the PBP/DBP interface, present in $AcrB^{WT}$-PAβN, are not retained.

Although small differences in the flexibility of the binding sites emerged from the comparison of the RMSFs of $AcrB^{G288D}$-PAβN and $AcrB^{WT}$-PAβN (both considered in the T state, Supplementary Fig. 13), higher hydration levels were detected in $AcrB^{G288D}$-PAβN within the DBP and particularly at residues around D288, which include F178 and adjacent residues in PN2. These findings are in good agreement with HDX-MS data (Fig. 4b).

A comparison of the binding poses in $AcrB^{G288D}$-PAβN (Supplementary Fig. 12) reveals a strong contribution to the stabilization of the system from stacking of the aromatic groups of the inhibitor with residues of the HT, in analogy to our findings in $AcrB^{WT}$-PAβN (Supplementary Fig. 6) and $AcrB^{WT}$-CIP-PAβN (Supplementary Fig. 9). Additional stabilization comes from cation-π interactions involving the guanidino group of PAβN and residues

belonging or proximal to the switch-loop, as well as the amino group of the inhibitor and F178 in Pose 1. Moreover, contacts are formed between PAβN and the substituted residue D288, involved (e.g. in Pose 1) in hydrogen bonds with the amino group of PAβN.

In both poses, stabilizing interactions further involve residues of the PC1/PC2 cleft, such as π -stacking with aromatic groups of PAβN (Pose 1, 2) or hydrogen bonds between the amino group of the inhibitor and the backbone of residues P669 and A670 (Pose 2) (see Supplementary Fig. 12).

From the analysis of the occupancy levels, it emerged that PAβN forms stable hydrogen bonds with residues D288 and the switch loop in Pose 1 and with residues S134 and I671 in Pose 2 (Supplementary Table 6). According to HDX-MS data, several among these residues belong to protected peptides (segments 611-629, 664-671). Moreover, peptide 291-300, adjacent to the peptide containing D288, is also protected. Taken together, these data suggest that interactions with PAβN may significantly contribute to the protection of part of the DBP, as reported for the WT protein.

AcrB^{G288D}-CIP-PAβN

As in AcrB^{WT}, CIP and PAβN are involved in direct interactions through hydrogen bonds that involve the amino group of the inhibitor and the carboxylic group of CIP (see Pose 1 in Supplementary Fig. 14 for a representation of the binding pose). The amino group of the inhibitor forms cation-π interactions with residue F178, while its guanidino group is oriented towards D288. Additional stabilization comes from the stacking of the aromatic groups of PAβN with the lower part of the HT (F136, Y327) and the cleft (segment 668-670). CIP is also implicated in stacking with residues close to the switch-loop (such as F615), as well as in interactions with hydrophobic residues proximal to the HT (I277, V612). In analogy to AcrB^{WT}-CIP-PAβN (Supplementary Fig. 9, Pose 1), therefore, contacts with the switch-loop are retained, but the interaction with the PBP/DBP interface is weakened. Moreover, stabilizing interactions also contribute some residues of the PC1/PC2 cleft representing the entering gate towards the PBP, as well as the mutated residue D288.

From the comparison of the flexibility and hydration properties of AcrB^{G288D}-CIP-PAβN and AcrB^{WT}-CIP-PAβN (Supplementary Fig. 15), it emerges that the switch-loop is considerably more rigid and dehydrated in the mutant. A net increase in hydration and flexibility is also detected for part of the PN2 portion of the DP (including segment 178-182, involved in interactions with the substrates). These data agree with the stabilization of the switch-loop and the increase in hydration of PN2 emerged from HDX-MS analyses (Fig. 4b).

As for AcrB^{WT}-CIP-PAβN, direct interactions between the two substrates that involve the carboxylic and carbonyl group of CIP are present in both the binding modes detected in AcrB^{G288D}-CIP-PAβN (Supplementary Table 8). In addition, π-stacking between one or both substrates and the HT and the switch loop were detected. Further stabilization is provided by cation-π interactions established between the amino group of PAβN and F178 (Pose 1). Importantly, D288 also contributes to stabilize the complex by forming high-occupancy hydrogen bonds with PAβN (Pose 1) or CIP (Pose 2) (Supplementary Table 7). Another common feature of both poses is the π-stacking formed with residues of the PC1/PC2 cleft. A major difference regards instead the interaction with the PBP/DBP interface, which is

indeed present only in Pose 2 and involves the phenylalanine and arginine moieties of PAβN and the carboxylic group of CIP. In Pose 1 PAβN is located inside the HT and CIP interacts with regions proximal to the switch loop and to the upper part of the DBP (including, for example, residues I277 and segment 178-182).

As for AcrB^{WT}-CIP, comparison between the binding poses of AcrB^{G288D}-CIP-PAβN and AcrB^{G288D}-CIP revealed that the co-presence of PAβN in the DBP determines a shift in the binding region of CIP (Supplementary Fig. 16). In the ternary complex, indeed, CIP tends to occupy the HT, interacting with residue D288 as well as with several hydrophobic residues like F136 and F628 (Supplementary Fig. 14). Less stabilizing interactions are formed with residues belonging to the upper part of the DBP, which instead form several contacts with CIP in the ternary complex. Like AcrB^{WT}-PAβN, analysis of hydrogen bonds occupancy revealed that both ligands establish very stable interactions with D288, both direct and water-mediated (Supplementary Table 7). Additional interactions are mainly formed with residues E152 and S180 in Pose 1, and with residues Q89, E130 and K131 in Pose 2 (Supplementary Fig. 14).

Supplementary Figures

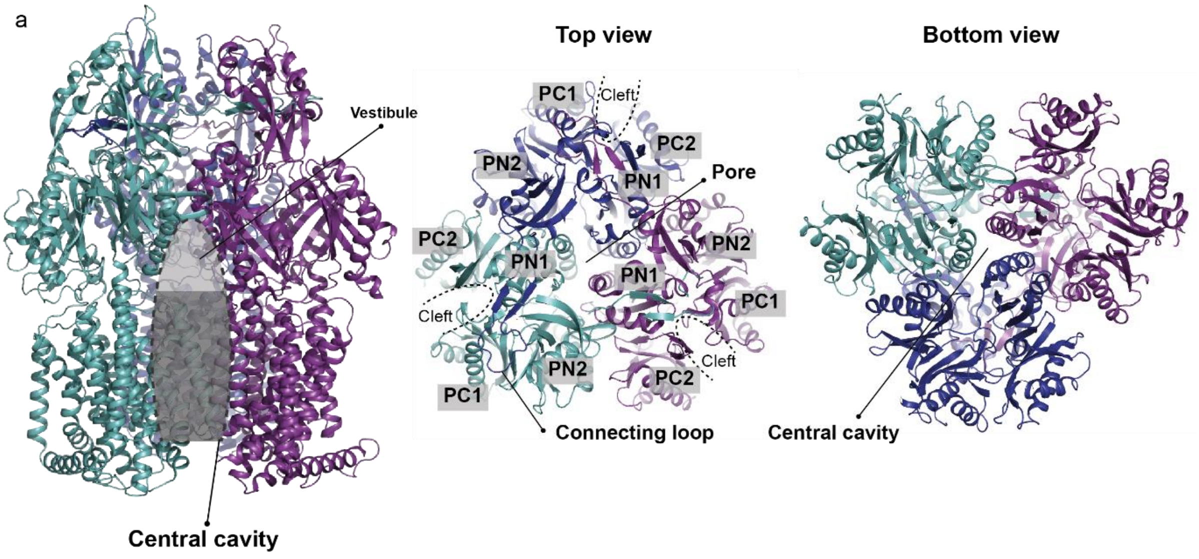

Supplementary Figure 1. AcrB central cavity and trimer architecture. The central cavity is shown by a dashed dotted line and a dark grey area, and the vestibule is shown by a light grey area.

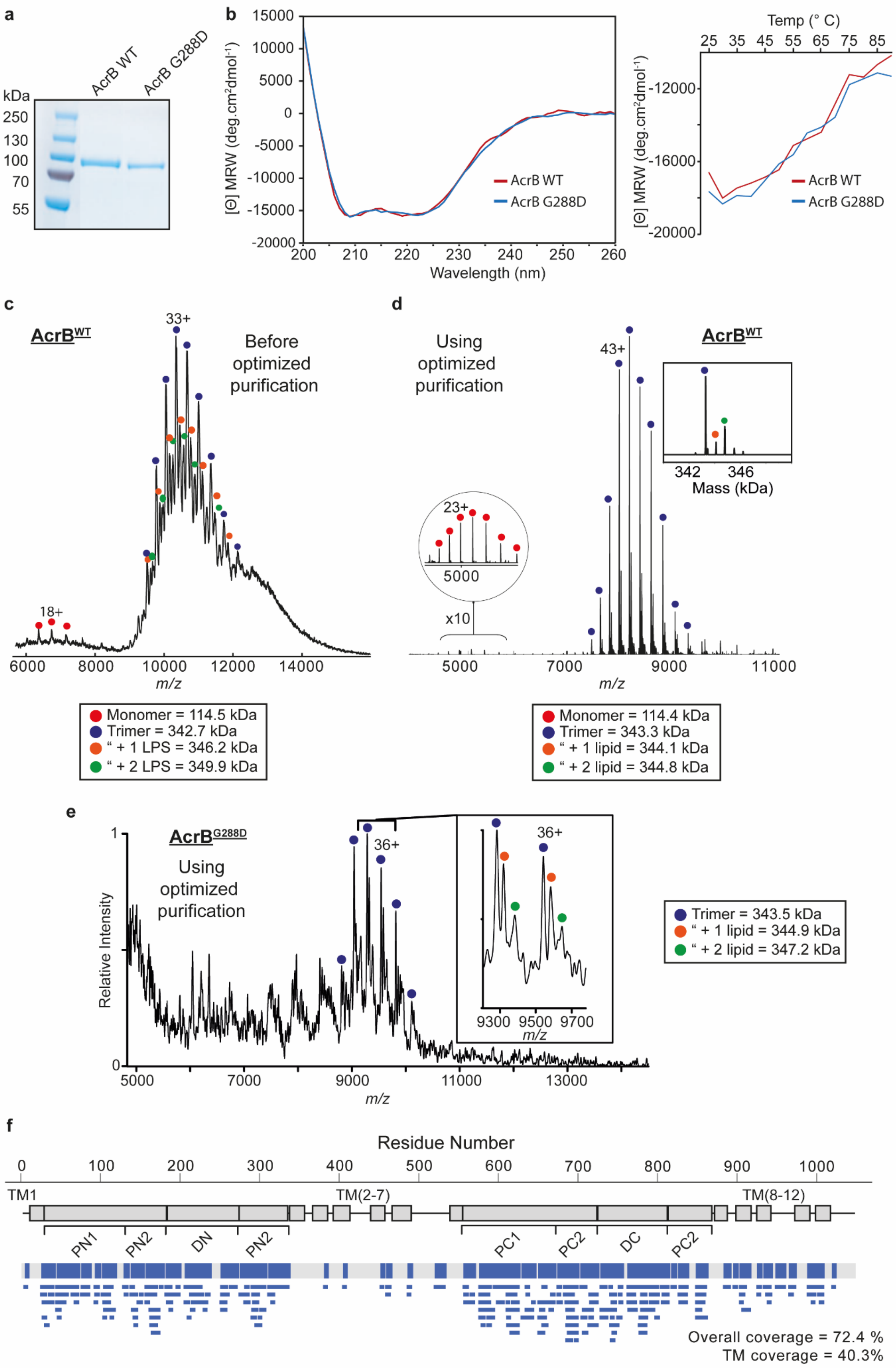
a
kDa
AcrB WT
AcrB G288D
250
130
100
70
55
b
Wavelength (nm)
[Θ] MRW (deg.cm²dmol⁻¹)
AcrB WT
AcrB G288D
Temp (° C)
c
AcrB^WT
Before optimized purification
33+
18+
m/z
Monomer = 114.5 kDa
Trimer = 342.7 kDa
" + 1 LPS = 346.2 kDa
" + 2 LPS = 349.9 kDa
d
Using optimized purification
AcrB^WT
43+
23+
x10
Mass (kDa)
m/z
Monomer = 114.4 kDa
Trimer = 343.3 kDa
" + 1 lipid = 344.1 kDa
" + 2 lipid = 344.8 kDa
e
AcrB^G288D
Using optimized purification
Relative Intensity
36+
m/z
Trimer = 343.5 kDa
" + 1 lipid = 344.9 kDa
" + 2 lipid = 347.2 kDa
f
Residue Number
TM1
TM(2-7)
TM(8-12)
PN1
PN2
DN
PN2
PC1
PC2
DC
PC2
Overall coverage = 72.4 %
TM coverage = 40.3%

Supplementary Figure 2. Biophysical characterization of AcrB^{WT} and AcrB^{G288D}. (**a**) SDS-PAGE of AcrB^{WT} and AcrB^{G288D} purified in DDM detergent micelles. Biological replicates of both AcrB^{WT} and AcrB^{G288D} (n = 3) were repeated independently with similar results. (**b**) Circular dichroism and thermal melt (as determined by the loss of the helical signature at 222 nm) of AcrB within DDM detergent micelles reveals that the WT and G288D mutant have similar average secondary structure and thermal stability. (**c**) Native mass spectra of AcrB before removal of detectable bound lipopolysaccharides (LPS) using optimized purification procedure as previously described (see Methods). LPS binding to membrane proteins during purification has been previously identified by additional masses of 3.2–3.7 kDa from the *apo* form[45]. Data was collected on a Synapt G2-Si mass spectrometer with AcrB^{WT} within Triton X-100 detergent micelles. (**d**) Native mass spectra of AcrB^{WT} within after optimized purification. Data was collected on a Thermo Scientific Q Exactive UHMR hybrid Quadrupole-orbitrap mass spectrometer with AcrB^{WT} within DDM detergent micelles. (**e**) Native mass spectra of AcrB^{G288D} after optimized purification. Data was collected on a Synapt G2-Si mass spectrometer with AcrB^{G288D} within Triton X-100 detergent micelles. (**f**) HDX-MS sequence coverage and peptide redundancy map of AcrB in DDM detergent micelles.

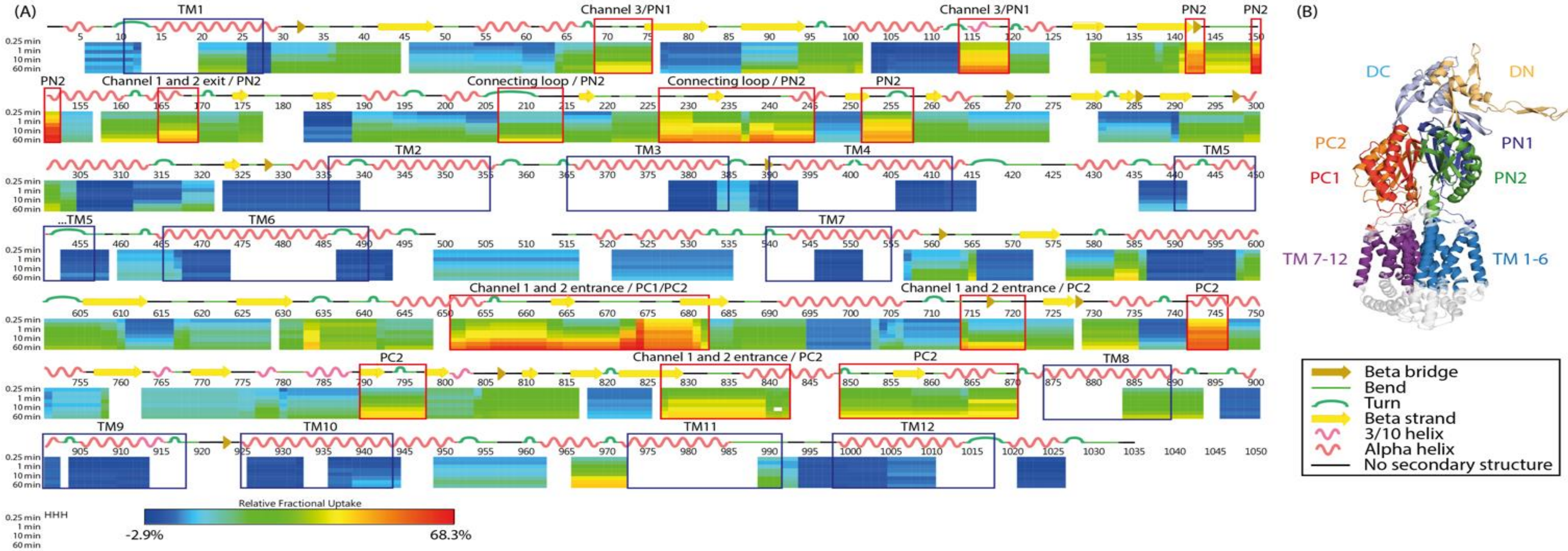

Supplementary Figure 3. HDX-MS heat maps representing the relative fractional uptake of deuterium for peptides of AcrB. The secondary structure of AcrB is shown above the peptide regions (key in box) and the subdomains of AcrB are shown on its monomer (right). The degree of relative fractional uptake of deuterium at each incubation time (from top to bottom: 15 s, 30s, 1 min, 3 min, 5 min, 10 min, 30 min and 60 min) are displayed according to the colour code shown. Uncoloured regions indicate areas with no peptide coverage. Red boxes annotate the regions that had the most significant change in uptake overtime. The secondary structure of AcrB is indicated above the heat map. All HDX-MS peptide data can be found in the Source Data file.

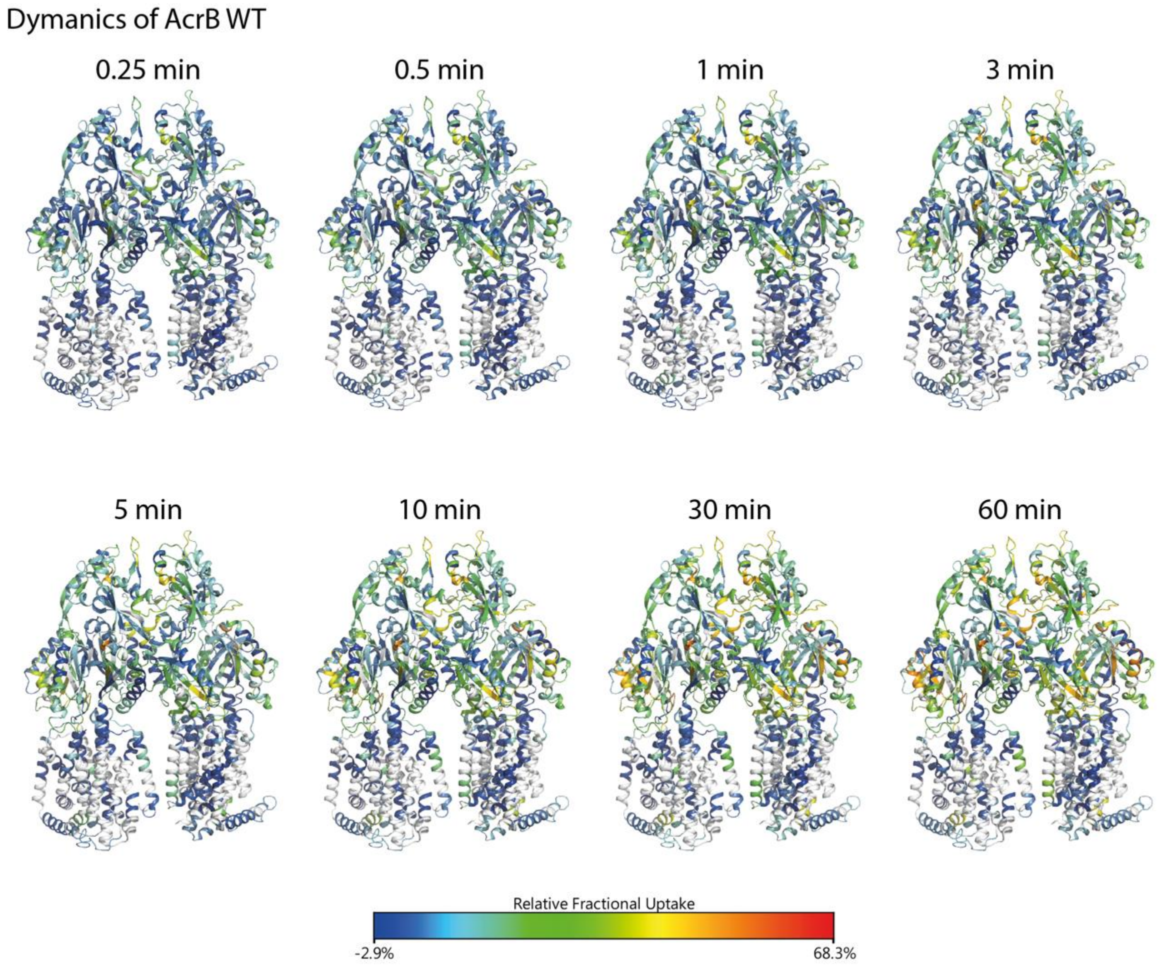

Supplementary Figure 4. HDX-MS heat maps from Supplementary Figure 3 translated onto the structure of AcrB^{WT} (PDB: 2HRT). A pseudo-dimer of AcrB is displayed for clarity.

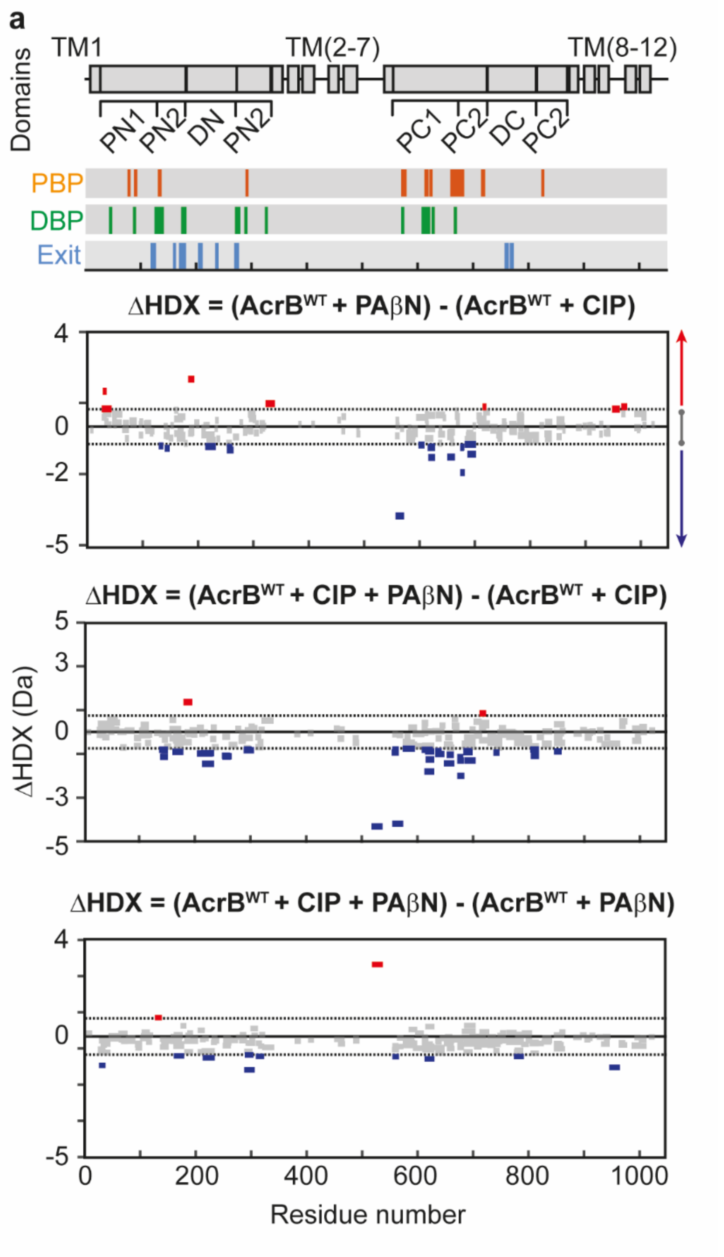

Supplementary Figure 5. Comparison of HDX-MS between AcrB^{WT} drug-binding conditions. Sum differential HDX (ΔHDX) plots for different drug conditions (ΔHDX = (AcrB^{WT}+drug(s)) – (AcrB^{WT}+drug(s)) for all time points collected. All data reported as in Fig. 2. All HDX-MS peptide data can be found in the Source Data file.

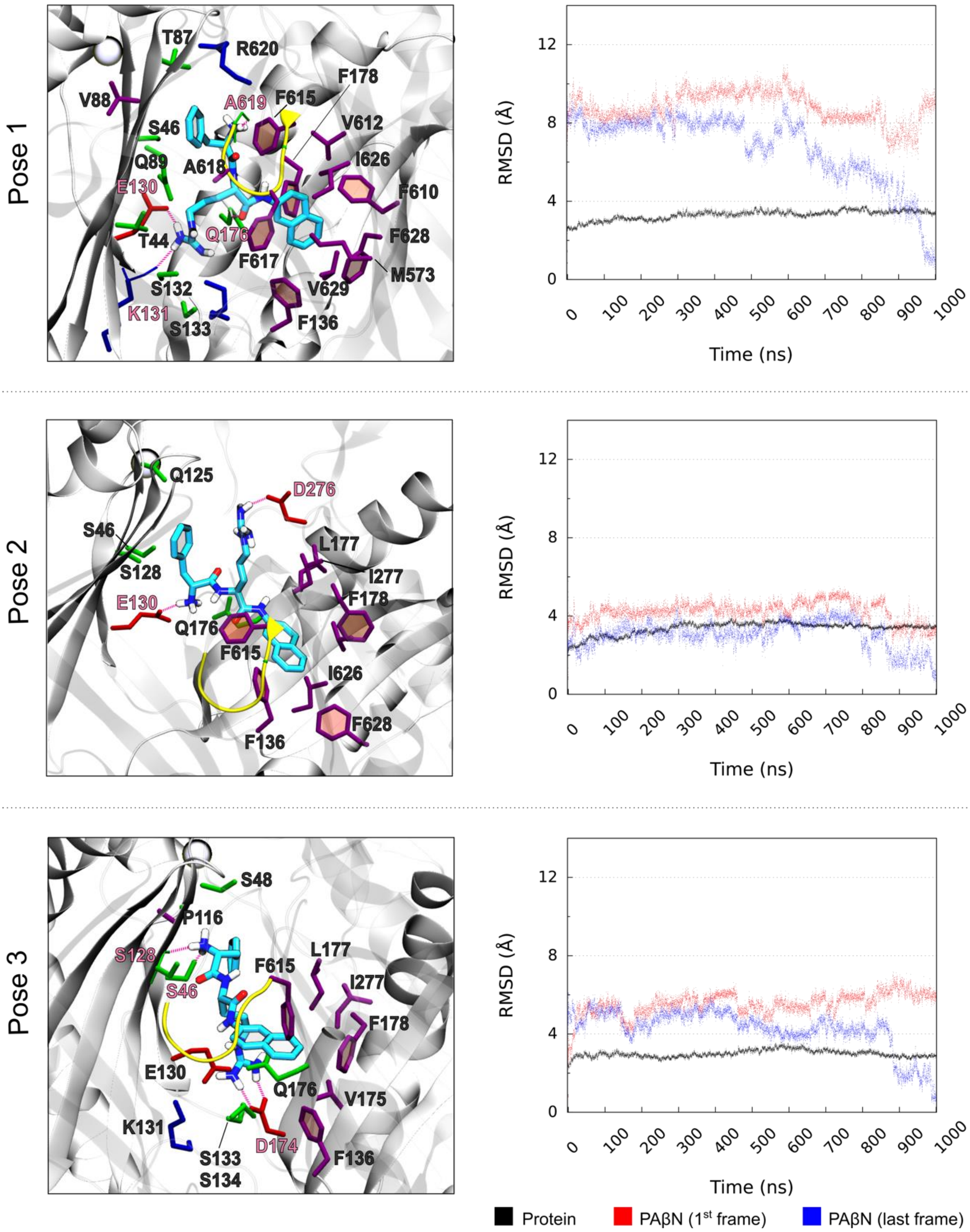

Supplementary Figure 6. Representative binding poses and RMSDs of AcrB^{WT}-PAβN (Pose 1 discussed in the main text). In the representation of the binding poses, PAβN is coloured by atom type (C atoms in cyan, N atoms in blue and oxygen atoms in red, H atoms in white – only polar H atoms are shown). Residues within 3.5 Å are also shown, coloured by residue type (red: acidic; blue: basic; green: polar; purple: hydrophobic). Hydrogen bonds formed by PAβN are highlighted through magenta sticks, and the involved residues are labelled in pink (see Supplementary Table 3 for high-occupancy hydrogen bonds involving PAβN). The switch-loop is shown in yellow and the C_α atoms of the residues Q124 and Y758 belonging to

the exit gate are represented as light blue spheres. Water molecules were not represented for clarity. See Supplementary Table 10 for the RMSD of each pose with respect to reference structure PDB:4U95.

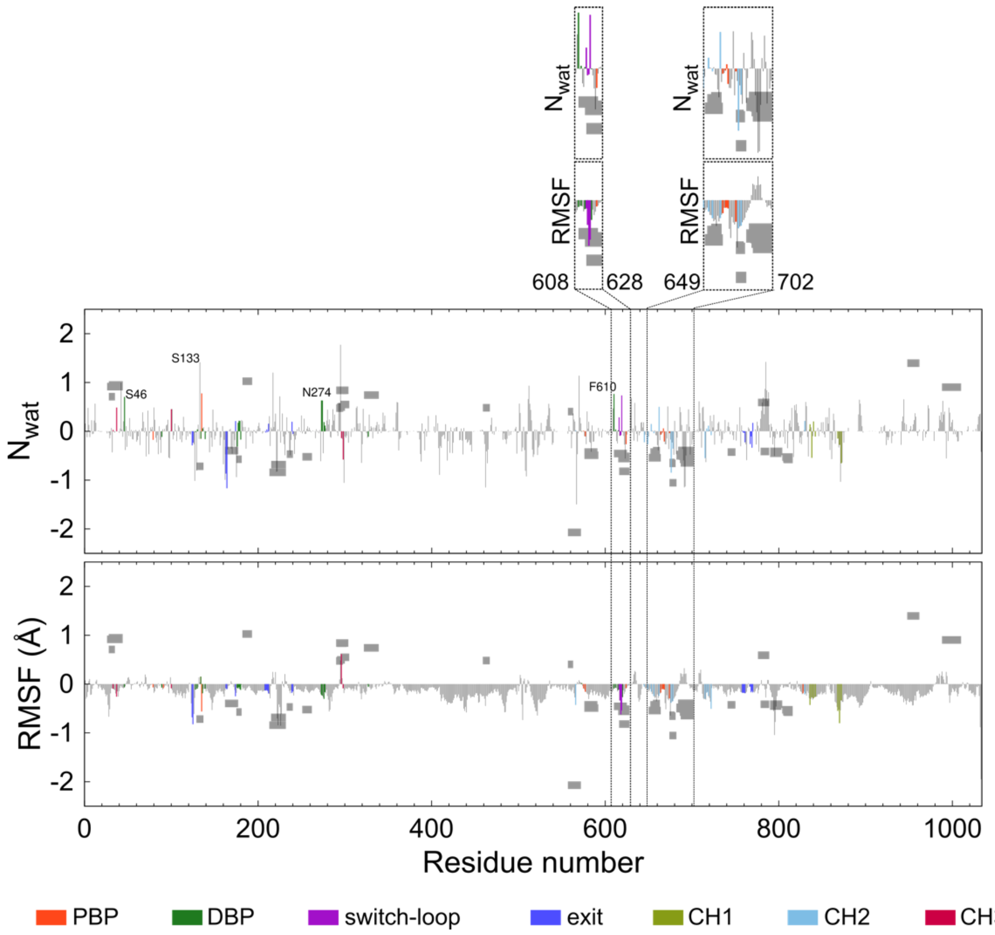

Supplementary Figure 7. Difference in first hydration shell (N_{wat}) and RMSF between AcrB^{WT}-PAβN and apo AcrB^{WT} (based on MD data from Pose 1 in Supplementary Fig. 6). Differences in N_{wat} and RMSF are represented as histograms, with regions directly involved in substrate transport highlighted in different colours (see Supplementary Table 1 for the definition of these regions). As a reference, HDX-MS data are represented as grey boxes (scale not shown). Both N_{wat} and RMSF differences have been computed between the T monomer of AcrB^{WT}-PAβN and the L monomer of apo AcrB^{WT} (see Methods). Regions of interest are highlighted in the upper part of the panel. In the N_{wat} plot, labelled residues are directly involved in interactions with PAβN and have a higher hydration in AcrB^{WT}-PAβN than in apo AcrB^{WT}.

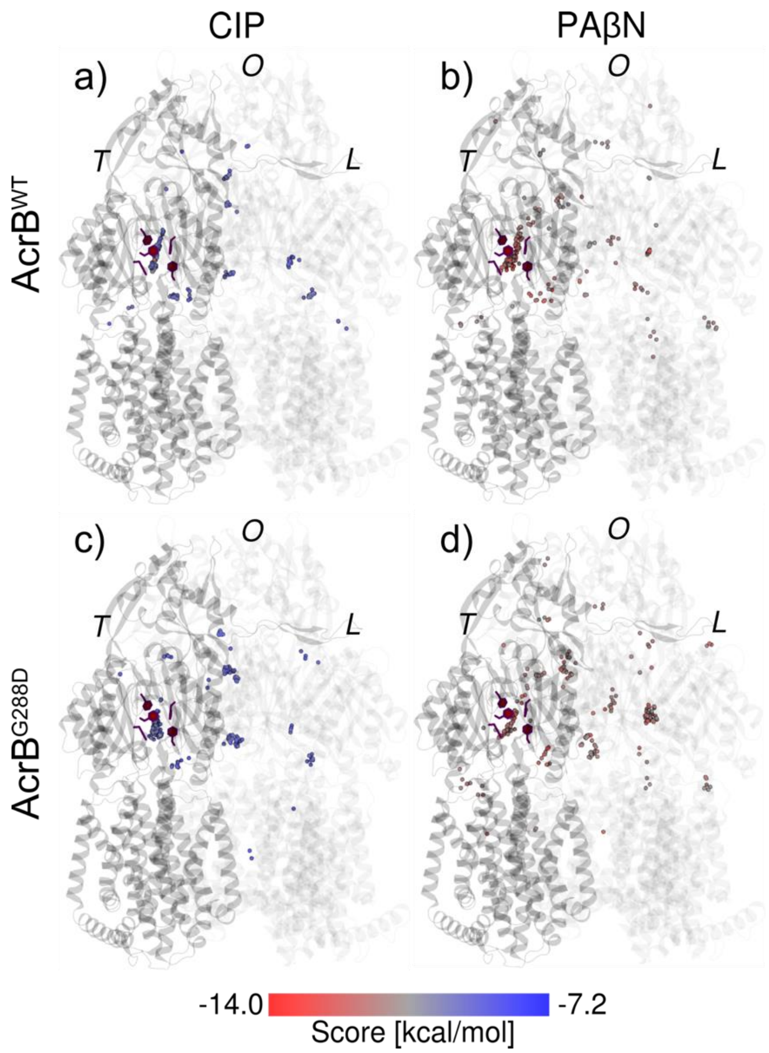

Supplementary Figure 8. Distribution of top 200 docking poses (only the centres of mass are shown for clarity) for CIP and PAβN onto AcrB^{WT} (a,b) and AcrB^{G288D} (c, d). The spheres are coloured according to the value of the (pseudo) free energy of binding (docking score). The monomers L, T and O are shown as transparent ribbons (T and O darkest and lightest, respectively). These distributions and the data in Supplementary Tables 2 and 5 justify our focus on the DBP of the T monomer. Details of the docking protocol are given in Methods.

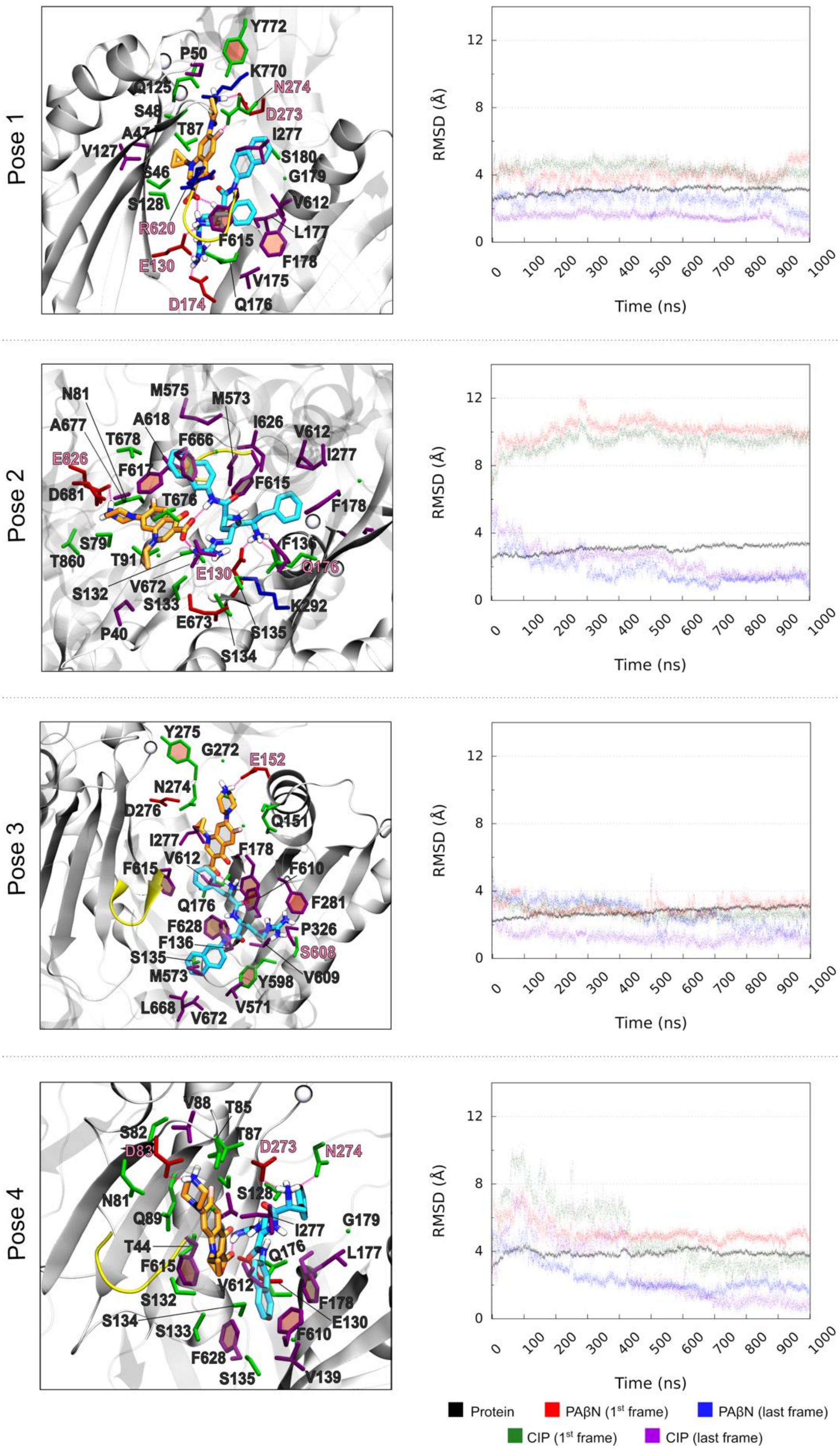

Supplementary Figure 9. Representative binding poses and RMSDs of AcrB^{WT}-CIP-PAβN (Pose 1 discussed in the main text). To distinguish between the inhibitor and antibiotic, carbon atoms of CIP and PAβN are coloured in orange and cyan, respectively. See: Supplementary Fig. 6 for further details; Supplementary Table 4 for a list of direct and

water-mediated hydrogen bonds established between each ligand and the protein; Supplementary Table 8 for high-occupancy hydrogen bonds between the ligands; Supplementary Table 10 for the RMSD of each pose with respect to reference structure PDB:4U95.

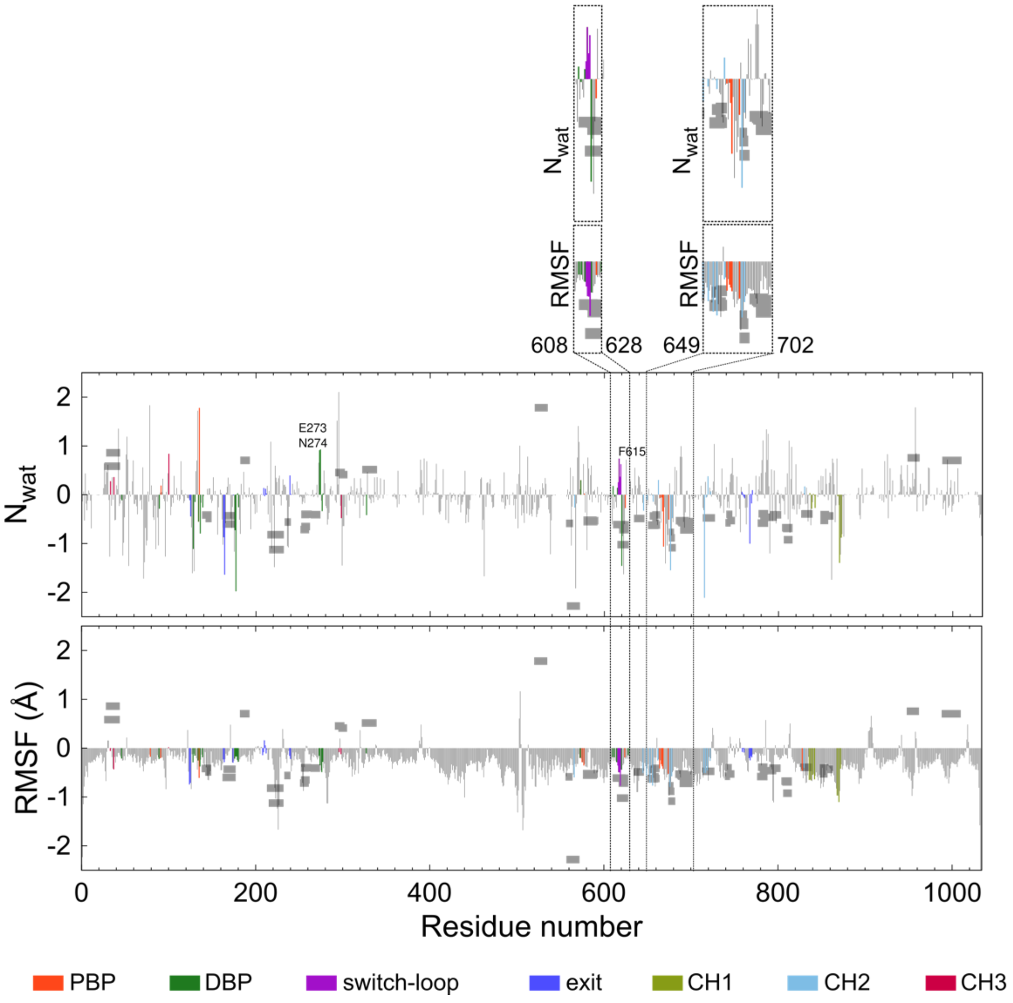

Supplementary Figure 10. Difference in first hydration shell (N_{wat}) and RMSF between AcrB^{WT}-CIP-PAβN and apo AcrB^{WT} (based on MD data from Pose 1 in Supplementary Fig. 9). Data is presented as in Supplementary Fig. 7.

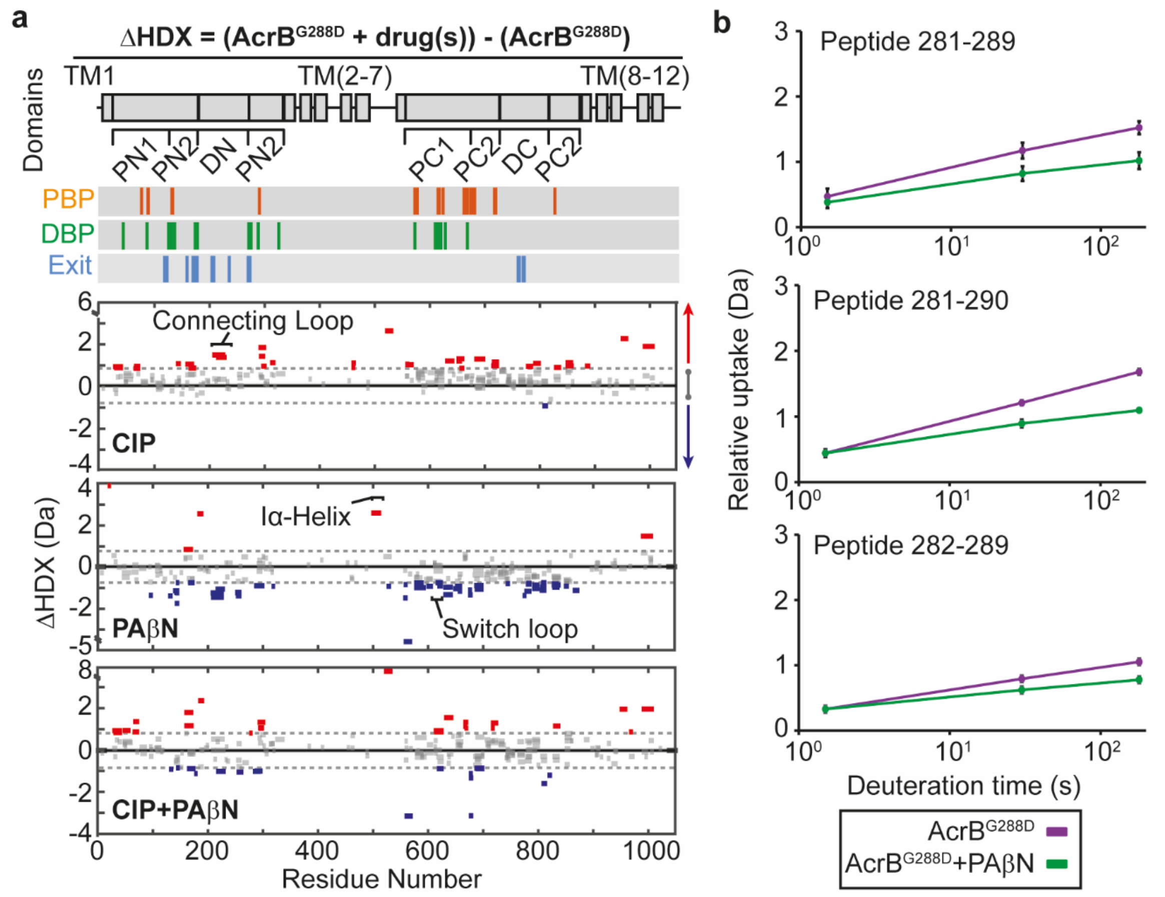

Supplementary Figure 11. Influence of drugs on AcrB^{G288D} structural dynamics. (**a**) Sum differential HDX (ΔHDX) plots for different drug conditions (ΔHDX = (AcrB^{G288D}+drug(s)) – AcrB^{G288D}) for all time points collected. (**b**) Deuterium uptake plots for D288 containing peptides, 281-289, 281-290 and 282-289 for (AcrB^{G288D}+PAβN) – AcrB^{G288D}. All data reported as in Fig. 2 and 4. All HDX-MS peptide data can be found in the Source Data file.

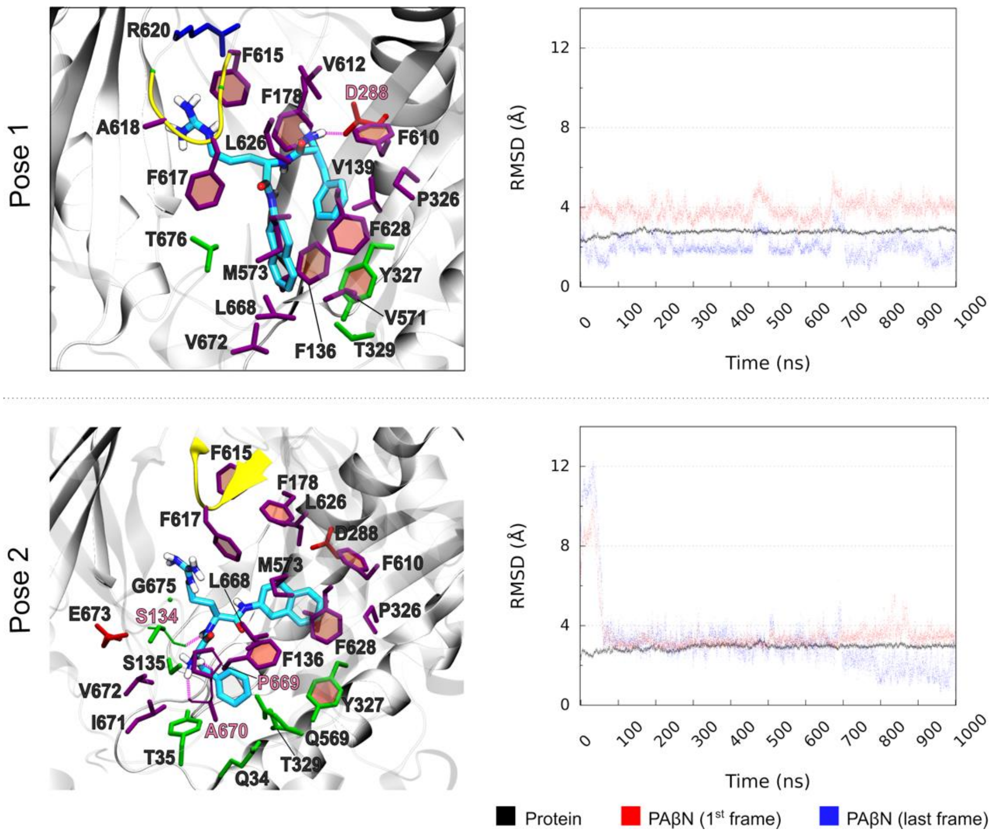

Supplementary Figure 12. Representative binding poses and RMSDs of AcrB^{G288D}-PAβN (Pose 1 discussed in the main text). Data presented as in Supplementary Fig. 6; see Supplementary Table 6 for a list of direct and water-mediated hydrogen bonds involving PAβN; Supplementary Table 10 for the RMSD of each pose with respect to reference structure PDB:4U95.

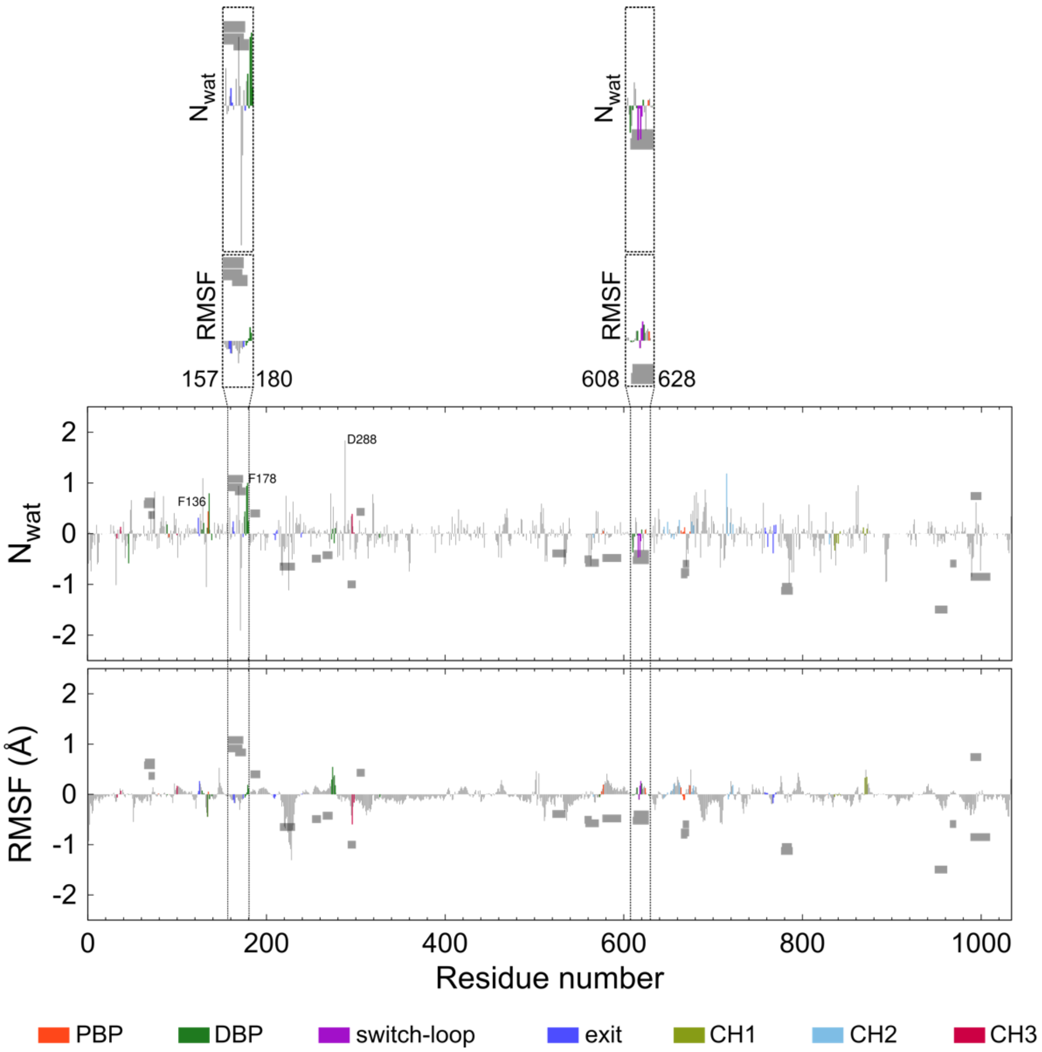

Supplementary Figure 13. Difference in first hydration shell (N_{wat}) and RMSF between AcrB^{G288D}-PAβN and AcrB^{WT}-PAβN (based on MD data from Pose 1 in Supplementary Fig. 12). Both AcrB^{G288D}-PAβN and AcrB^{WT}-PAβN were considered in the T state (see Methods). Data presented as in Supplementary Fig. 7.

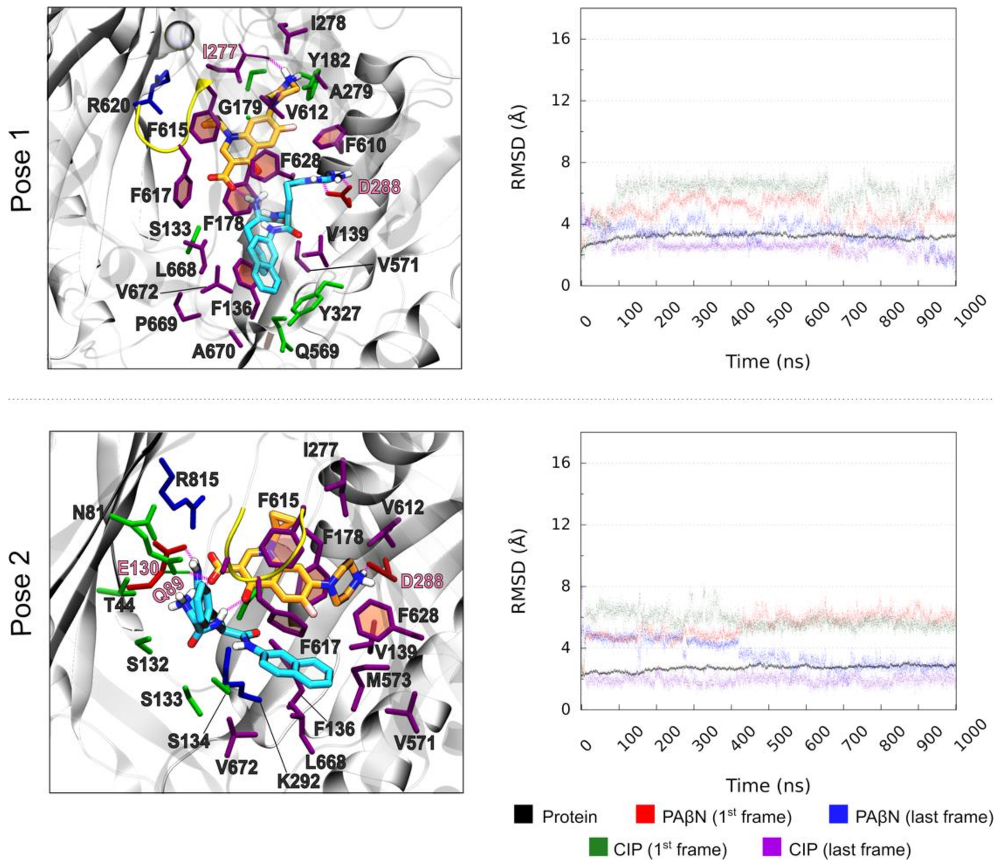

Supplementary Figure 14. Representative binding poses and RMSDs of AcrB^{G288D}-CIP-PAβN (Pose 1 discussed in the main text). Data presented as in Supplementary Fig. 6; see Supplementary Table 7 for a list of direct and water-mediated hydrogen bonds established by each ligand with AcrB; Supplementary Table 8 for high-occupancy hydrogen bonds between the ligands; Supplementary Table 10 for the RMSD of each pose with respect to reference structure PDB:4U95.

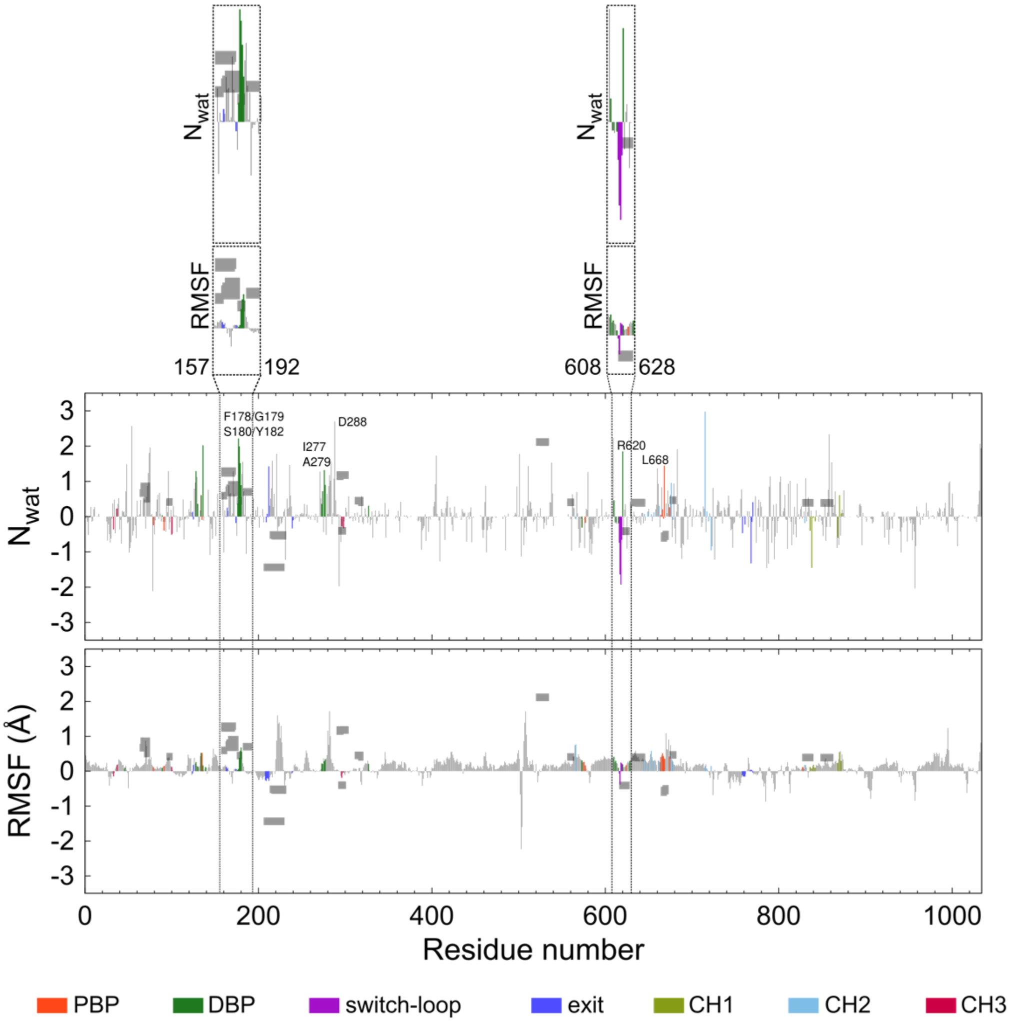

Supplementary Figure 15. Difference in first hydration shell (N_{wat}) and RMSF between AcrB^{G288D}-CIP-PAβN and apo AcrB^{WT}-CIP-PAβN (based on MD data from Pose 1 in Supplementary Fig. 14). Both AcrB^{G288D}-CIP-PAβN and AcrB^{WT}-CIP-PAβN were considered in the T state (see Methods). Data presented as in Supplementary Fig. 7.

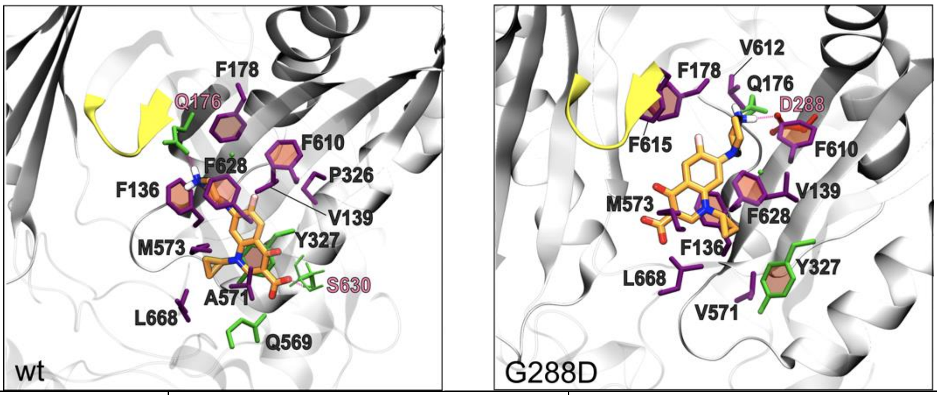

System	H-bonds		Water-mediated interactions	
	Residue	Occupancy (%)	Residue	Occupancy (%)
AcrB^{WT}	Q176	41	G570	27
AcrB^{G288D}	D288	100	-	-
	F178	41		
	Q176	33		

Supplementary Figure 16. Representative binding poses of AcrB^{WT}-CIP and AcrB^{G288D}-CIP (upper panel) and high-occupancy hydrogen bonds (H-bonds) established between CIP and the protein (lower table). See Supplementary Fig. 6 for further details on the representation of the binding poses. As stated in the Supplementary Methods, the binding pose of AcrB^{WT}-CIP features the substrate within the same region as in Ref. [Vargiu and Nikaido, PNAS, 2012], although the orientation is opposite. Hydrogen bond analyses were performed on the last 300 ns of each simulation (see Methods). Only direct or water-mediated bonds with occupancy higher than 20% have been reported.

Supplementary Tables

Regions	Residues
Central cavity	25-33, 36, 37, 96, 97, 385-389, 457-466, 468, 469
Distal binding pocket (DBP)	44, 46, 48, 87, 89, 128, 130, 132, 134, 136, 139, 176--180, 273, 274, 276, 277, 327, 573, 610, 612, 615, 617, 620, 626, 628
Hydrophobic trap (HT)	136, 178, 289, 291, 573, 610, 612, 615, 626, 628
Proximal binding pocket (PBP)	79-81, 89-91, 132-134, 573, 575, 577, 617, 662-669, 672-681, 683, 717, 719, 815, 826, 828-830
Connecting loop	206-243
Iα-Helix	520-534
Switch loop	615-620
Exit gate (EG)	124, 125, 163, 164, 174, 208-221, 239, 240, 758-761, 767-770
Channel 1 (CH1)	836, 838, 840, 842, 868, 870, 872
Channel 2 (CH2)	566, 645, 649, 653, 656, 662, 676, 678, 715, 717, 719, 722, 830
Channel 3 (CH3)	33, 37, 100, 296, 298

Supplementary Table 1. List of peptides considered in the regions of AcrB.

	%res	30%			40%		
	Site	N	ΔG_{max}	<ΔG>	N	ΔG_{max}	<ΔG>
PAβN	PBP_L	12	-11.5	-10.9 ± 0.3	3	-11.3	-10.9 ± 0.3
	PBP_T	19	-13.0	-11.6 ± 0.6	-	-	-
	$CH1_L$	1	-10.8	-10.8	1	-10.8	-10.8
	$CH1_T$	-	-	-	-	-	-
	$CH2_L$	1	-11.3	-11.3	-	-	-
	$CH2_T$	-	-	-	-	-	-
	$CH3_L$	15	-12.3	-11.3 ± 0.4	9	-12.3	-11.4 ± 0.4
	$CH3_T$	33	-12.5	-11.4 ± 0.4	22	-12.5	-11.5 ± 0.4
	DBP_T	148	-13.7	-11.6 ± 0.7	87	-13.7	-11.7 ± 0.7
CIP	PBP_L	-	-	-	-	-	-
	PBP_T	-	-	-	-	-	-
	$CH1_L$	-	-	-	-	-	-
	$CH1_T$	-	-	-	-	-	-
	$CH2_L$	-	-	-	-	-	-
	$CH2_T$	-	-	-	-	-	-
	$CH3_L$	55	-10.2	-9.4 ± 0.3	19	-9.6	-9.3 ± 0.2
	$CH3_T$	31	-10.0	-9.5 ± 0.2	4	-9.7	-9.4 ± 0.3
	DBP_T	123	-11.5	-9.7 ± 0.4	20	-10.3	-9.6 ± 0.4

Supplementary Table 2. Number of poses, maximum and average (pseudo) free energy of CIP and PAβN binding to AcrB^{WT}. Binding in PBP_L, PBP_T, $CH1_L$, $CH1_T$, $CH2_L$, $CH2_T$, $CH3_L$, $CH3_T$ and DBP_T as obtained from blind ensemble docking (for each compound we docked 10 conformations onto 10 conformations of the protein, see Methods for details). The percentages in the first row are meant to identify the poses having contacts (that is minimum ligand-residue distance below a cutoff – here set to 3.5 Å) at least with 30% or 40% of residues lining the corresponding site. From blind docking calculations it is clear that, overall, there is a significant overlap between the poses of PAβN and CIP (see Supplementary Fig. 8a,b). Both compounds accumulate high-affinity binding poses within the DBP of monomer T (DBP_T). It is also evident how the inhibitor has a larger binding affinity than CIP towards that pocket. However, other poses for PAβN are found at $CH1_{L/T}$, $CH2_L$, and $CH3_{L/T}$, as well as within $PBP_{L/T}$. Regarding CIP, we also see poses just beneath the CH3 of monomers L and T and farther away from CH1 of monomer T, while as expected no poses are found within the PBP. These data suggest that PAβN, whose affinities at $CH3_{L/T}$ are comparable to those at DBP_T, could in principle compete with CIP also during the uptake by AcrB, thus not only regarding binding at the preferred site.

Ligand	Pose	H-bonds		Water-mediated interactions	
		Residue	Occupancy (%)	Residue	Occupancy (%)
PAβN	1	Q176	67	E673	39
		G616	57	F617	36
		E673	40	S133	21
		G619	37		
		F617	23		
	2	D276	99	E130	67
		E130	87	D276	53
		Q176	60	L177	50
		G614	24		
	3	E273	100	D276	79
		S46	90	E273	45
		S48	39	Q176	26
		D174	26		

Supplementary Table 3. Intermolecular hydrogen bonds (H-bonds) and water-mediated interactions involving PAβN in AcrB^{WT}-PAβN (based on MD simulations). Analyses have been conducted on the last 300 ns of each simulation (see Methods). Only interactions with occupancy higher than 20% have been reported. Representative poses are shown in Supplementary Fig. 6.

Ligand	Pose	H-bonds		Water-mediated interactions	
		Residue	Occupancy (%)	Residue	Occupancy (%)
PAβN	1	E130	100	D174	71
		D174	100	E130	57
		L177	69	Q89	42
	2	Q176	76	E130	40
		E130	66		
		S132	60		
		S133	45		
		S134	23		
		V672	20		
	3			S608	41
	4	D276	100	E273	45
		N274	69	D276	50
		S128	51	L177	43
		L177	48	S128	27
		S46	47		
CIP	1	R620	100	Q89	100
		E273	94	S128	100
		Q125	84	D681	40
	2	T676	90	E826	22
		E826	69		
		Y77	41		
	3	E152	94	E152	46
		Q176	80		
		N274	60		
	4	D83	100	L177	43
		Q176	58		
		T44	51		
		S133	51		
		T87	46		

Supplementary Table 4. Intermolecular hydrogen bonds (H-bonds) and water-mediated interactions involving ligands in AcrB^{WT}-CIP-PAβN (based on MD simulations). See Supplementary Table 3 for details and Supplementary Figure 9 for representative binding poses.

	%res	30%			40%		
	**Site**	**N**	**ΔG_{max}**	**<ΔG>**	**N**	**ΔG_{max}**	**<ΔG>**
**PAβN**	**PBP_L**	-	-	-	-	-	-
	**PBP_T**	3	-11.5	-11.1 ± 0.3	-	-	-
	**$CH1_L$**	-	-	-	-	-	-
	**$CH1_T$**	2	-10.8	-10.8 ± 0.0	2	-10.8	-10.8 ± 0.0
	**$CH2_L$**	-	-	-	-	-	-
	**$CH2_T$**	2	-11.1	-11.1 ± 0.0	-	-	-
	**$CH3_L$**	18	-12.9	-11.5 ± 0.6	7	-12.0	-11.5 ± 0.4
	**$CH3_T$**	11	-13.5	-11.7 ± 0.8	4	-11.8	-11.3 ± 0.3
	**DBP_T**	24	-13.4	-11.6 ± 0.7	2	-11.3	-11.2 ± 0.1
**CIP**	**PBP_L**	-	-	-	-	-	-
	**PBP_T**	-	-	-	-	-	-
	**$CH1_L$**	-	-	-	-	-	-
	**$CH1_T$**	-	-	-	-	-	-
	**$CH2_L$**	-	-	-	-	-	-
	**$CH2_T$**	-	-	-	-	-	-
	**$CH3_L$**	14	-9.9	-9.2 ± 0.3	-	-	-
	**$CH3_T$**	9	-9.4	-9.1 ± 0.2	-	-	-
	**DBP_T**	5	-9.8	-9.5 ± 0.2	1	-9.3	-9.3 ± 0.0

Supplementary Table 5. Number of poses, maximum and average (pseudo)free-energy of CIP and PAβN binding to $AcrB^{G288D}$. Binding in PBP_L, PBP_T, $CH1_L$, $CH1_T$, $CH2_L$, $CH2_T$, $CH3_L$, $CH3_T$ and DBP_T as obtained from blind ensemble docking (for each compound we docked 10 conformations onto 10 conformations of the protein, see caption of Supplementary Table 2 for further details and Supporting Fig. 8c,d for the distribution of the poses).

Ligand	Pose	H-bonds		Water-mediated interactions	
		Residue	Occupancy (%)	Residue	Occupancy (%)
PAβN	1	D288	100	D288	65
		G616	82	Q176	27
		F617	21		
	2	S134	95		
		I671	67		

Supplementary Table 6. Intermolecular hydrogen bonds (H-bonds) and water-mediated interactions involving PAβN in AcrB^{G288D}-PAβN (MDs data). See Supplementary Table 3 for details and Supplementary Figure 12 for representative binding poses.

Ligand	Pose	H-bonds		Water-mediated interactions	
		Residue	Occupancy (%)	Residue	Occupancy (%)
PAβN	1	D288	100	D288	100
	2	E130	94	E130	68
				K131	67
				T91	64
				Q89	31
CIP	1	I277	32	E152	41
		S180	31		
		D276	23		
	2				
	3	E152	94	E152	46
		Q176	80		
		N274	60		
	4	D83	100	L177	43
		Q176	58		
		T44	51		
		S133	51		
		T87	46		

Supplementary Table 7. Intermolecular hydrogen bonds (H-bonds) and water-mediated interactions involving ligands in AcrB^{G288D}-CIP-PAβN (MDs data). See Supplementary Table 3 for details and Supplementary Figure 14 for representative binding poses.

System	Pose	Involved groups		Occupancy (%)
		CIP	PAβN	
AcrB^{WT}-CIP-PAβN	1	CO	NH_3^+ (Phe)	86
		CO	NH (Phe)	86
	2	CO_2	NH (β-napht.)	62
		CO	NH (β-napht.)	47
		CO	NH_2 (Arg)	55
	3	CO	NH (Phe)	79
	4	CO	NH (Arg)	32
AcrB^{G288D}-CIP-PAβN	1	CO	NH (Phe)	57
		CO	NH (Phe)	30
	2	CO_2	NH_3^+ (Phe)	93
		CO	NH (Arg)	68
		CO	NH_2 (Arg)	43

Supplementary Table 8. Occupancies of intermolecular hydrogen bonds between the ligands in AcrB^{WT}-CIP-PAβN and AcrB^{G288D}-CIP-PAβN. For a better identification of the functional groups of PAβN, their moieties of belonging (Phe, Arg and β-napthilamide) have been indicated in parentheses. Analyses have been conducted on the last 300 ns of each simulation (see Methods). Only interactions with occupancy higher than 20% have been reported.

Name	Sequence (5'-3')	Use
TEV_sGFP_del_Fwd	CACCACCACCACCACCAC	Deletion of TEV-sGFP region using Q5 mutagenesis kit from pET15b-AcrB-sGFP-6xHis plasmid from Reading *et al.*[44]; forward primer
TEV_sGFP_del_Rev	ATGATGATCGACAGTATGGCTG	Deletion of TEV-sGFP region using Q5 mutagenesis kit from pET15b-AcrB-sGFP-6xHis plasmid from Reading *et al.*[44]; reverse primer
pBRforEco	AATAGGCGTATCACGAGGC	For pBR322 plasmid sequencing: upstream of EcoRI site; forward primer
pBRrevBam	GGTGATGTCGGCGATATAGG	For pBR322 plasmid sequencing: downstream of BamHI; reverse primer
acrAB_EcoRI_Fwd	TCGTCTTCAAGAATTCGTTGGCGCGTTTCTTGCG	Cloning *acrAB* genes with its natural promoter, including the 'marbox' sequence, from K-12 *Escherichia coli* chromosomal DNA into pBR322 (linearized with HindIII and EcoRI restriction enzymes); forward primer
acrAB_His_HindIII_Rev	TACCGCATTAAAGCTTTCAATGATGATGATGATGATGATGATGATCGACAGTATGGCTGTG	As above but, a 6xHistidine tag sequence was included in the reverse primer to provide a 6xHis tag at the C-terminus of AcrB; reverse primer
AcrB_G288D_Q5_Fwd	ACCGGCTTCCgatCTGGGGATCA	Q5 mutagenesis of G288D in *acrB* gene, forward primer
AcrB_G288D_Q5_Rev	TGGCCGTTAAACTCTGCGATGATG	Q5 mutagenesis of G288D in *acrB* gene, reverse primer

Supplementary Table 9. **Primers used for plasmid construction** (see Methods section for more details).

<table>
<tr><th rowspan="2">System</th><th rowspan="2">Pose</th><th colspan="2">RMSD (Å)</th></tr>
<tr><th>T monomer</th><th>T monomer:
PBP, DBP, switch loop</th></tr>
<tr><td rowspan="3">AcrB^{WT}-PAβN</td><td>1</td><td>2.5</td><td>2.2</td></tr>
<tr><td>2</td><td>3.2</td><td>3.3</td></tr>
<tr><td>3</td><td>2.9</td><td>2.9</td></tr>
<tr><td rowspan="4">AcrB^{WT}-CIP-PAβN</td><td>1</td><td>2.7</td><td>2.0</td></tr>
<tr><td>2</td><td>2.7</td><td>2.2</td></tr>
<tr><td>3</td><td>2.6</td><td>2.3</td></tr>
<tr><td>4</td><td>3.2</td><td>2.3</td></tr>
<tr><td rowspan="2">AcrB^{G288D}-PAβN</td><td>1</td><td>2.5</td><td>2.5</td></tr>
<tr><td>2</td><td>2.8</td><td>2.1</td></tr>
<tr><td rowspan="2">AcrB^{G288D}-CIP-PAβN</td><td>1</td><td>2.8</td><td>2.6</td></tr>
<tr><td>2</td><td>2.3</td><td>1.9</td></tr>
</table>

Supplementary Table 10. Backbone RMSD of each pose with respect to the X-ray crystal structure 4U95 of *E. coli* AcrB (resolution: 2.0 Å). Calculations were performed on the T monomer of the protein (residues 1-1033) and on a sub-selection composed by the PBP, DBP and the switch loop (see Supplementary Table 1 for a definition of these regions). For each pose, the RMSD was computed on the centre of the representative cluster of the last 300 ns of MD simulation.

System	Reference
AcrB^{WT}-PAβN (T state)	Apo AcrB^{WT} (L state)
AcrB^{WT}-CIP-PAβN (T state)	Apo AcrB^{WT} (L state)
AcrB^{G288D}-PAβN (T state)	AcrB^{WT}-PAβN (T state)
AcrB^{G288D}-CIP-PAβN (T state)	AcrB^{G288D}-CIP-PAβN (T state)

Supplementary Table 11. Systems considered for the analyses of flexibility and hydration properties (based on MD simulations), and respective reference structures. The state of each system was chosen in agreement with Wang et al.[16] (see Methods).
