# Supplementary material for: Perturbed structural dynamics underlie inhibition and altered efflux of the multidrug resistance pump AcrB
Source: Nat Commun. 2020 Nov 4;11:5565. doi: 10.1038/s41467-020-19397-2 (PMC7642415; doi:10.1038/s41467-020-19397-2)
Supplement: Supplementary file 7 — Reporting Summary [file 41467_2020_19397_MOESM7_ESM.pdf]

nature research

Corresponding author(s): Eamonn Reading, Argyris Politis

Last updated by author(s): Oct 7, 2020

# Reporting Summary

Nature Research wishes to improve the reproducibility of the work that we publish. This form provides structure for consistency and transparency in reporting. For further information on Nature Research policies, see our Editorial Policies and the Editorial Policy Checklist.

## Statistics

For all statistical analyses, confirm that the following items are present in the figure legend, table legend, main text, or Methods section.

| n/a | Confirmed | |
|---|---|---|
| ☐ | ☒ | The exact sample size (*n*) for each experimental group/condition, given as a discrete number and unit of measurement |
| ☐ | ☒ | A statement on whether measurements were taken from distinct samples or whether the same sample was measured repeatedly |
| ☐ | ☒ | The statistical test(s) used AND whether they are one- or two-sided<br>*Only common tests should be described solely by name; describe more complex techniques in the Methods section.* |
| ☒ | ☐ | A description of all covariates tested |
| ☐ | ☒ | A description of any assumptions or corrections, such as tests of normality and adjustment for multiple comparisons |
| ☐ | ☒ | A full description of the statistical parameters including central tendency (e.g. means) or other basic estimates (e.g. regression coefficient) AND variation (e.g. standard deviation) or associated estimates of uncertainty (e.g. confidence intervals) |
| ☐ | ☒ | For null hypothesis testing, the test statistic (e.g. *F*, *t*, *r*) with confidence intervals, effect sizes, degrees of freedom and *P* value noted<br>*Give P values as exact values whenever suitable.* |
| ☒ | ☐ | For Bayesian analysis, information on the choice of priors and Markov chain Monte Carlo settings |
| ☒ | ☐ | For hierarchical and complex designs, identification of the appropriate level for tests and full reporting of outcomes |
| ☒ | ☐ | Estimates of effect sizes (e.g. Cohen's *d*, Pearson's *r*), indicating how they were calculated |

*Our web collection on statistics for biologists contains articles on many of the points above.*

## Software and code

Policy information about availability of computer code

| | |
|---|---|
| Data collection | MassLynx (v. 4.1, Waters) was used for controlling the Synapt G2-Si mass spectrometer for the acquisition of MS and MS/MS data. Xcalibur software (v. 4.3, Thermo Fisher Scientific) was used for controlling the Thermo Scientific Q Exactive UHMR hybrid Quadrupole-orbitrap mass spectrometer for the acquisition of MS and MS/MS data. |
| Data analysis | For HDX-MS data: Sequence identification was performed from MSE data of digested undeuterated samples using the ProteinLynx Global Server 2.5.1 software (Waters). Peptides were filtered and HDX analysed using DynamX (v. 3.0, Waters). Deuteros software (v. 1.0) was used for the statistical analysis and plotting of HDX-MS data (available at https://github.com/andymlau/Deuteros). For Native MS data: MassLynx (v. 4.1, Waters) or Xcalibur (v. 4.3) and Biopharma software (v. 3.1) (both Thermo Fisher Scientific) were used to assign and analyze native mass spectrometry data. Fluorescence Polarization binding: Data was fit using ORIGIN Ver. 7.5. (OriginLab Corporation, Northampton, MA, USA). Molecular docking: Performed using Autodock Vina. Molecular dynamics simulations: All-atom molecular dynamics (MD) simulations performed with the AMBER18 package. MD trajectories were analyzed using either in-house tcl and bash scripts or the cpptraj tool of AMBER18. Figures were prepared using gnuplot 5.0 and VMD 1.9.3. |

For manuscripts utilizing custom algorithms or software that are central to the research but not yet described in published literature, software must be made available to editors and reviewers. We strongly encourage code deposition in a community repository (e.g. GitHub). See the Nature Research guidelines for submitting code & software for further information.

## Data

Policy information about availability of data

All manuscripts must include a data availability statement. This statement should provide the following information, where applicable:

- Accession codes, unique identifiers, or web links for publicly available datasets
- A list of figures that have associated raw data
- A description of any restrictions on data availability

HDX-MS data and meta-data supporting the findings are published in the Source Data File and the Supporting Data Tables respectively, in line with suggestions made by the HDX-MS 'white paper' (Masson, G., et al., Nature Methods, 16, 595–602, (2019)). HDX-MS proteomics data files including processed DynamX files have been deposited to the ProteomeXchange Consortium via the PRIDE partner repository with the project accession code: PXD019047 [http://www.ebi.ac.uk/pride/archive/projects/PXD019047]. Upon request, we will share the trajectories generated by our MD simulations: As they occupy almost 4TB of data, we believe this the most suitable option to make our data available to interested readers. The trajectories will be available anyone at any time by sending an e-mail to Attilio Vittorio Vargiu (vargiu@dsf.unica.it). The source data underlying Figs. 2a-b, 3a-b, 4b, 5b-c, and Supplementary Figures 3, 4, 5 and 11 are provided as a Source Data file with this paper.

# Field-specific reporting

Please select the one below that is the best fit for your research. If you are not sure, read the appropriate sections before making your selection.

☒ Life sciences ☐ Behavioural & social sciences ☐ Ecological, evolutionary & environmental sciences

For a reference copy of the document with all sections, see nature.com/documents/nr-reporting-summary-flat.pdf

# Life sciences study design

All studies must disclose on these points even when the disclosure is negative.

| | |
|---|---|
| Sample size | Samples were measured in singlets with selected measuring points being measured in at least triplicate (n = 3). |
| Data exclusions | Peptides were excluded if they were insufficiently fragmented and/or if the mass error was above 15 ppm. |
| Replication | All deuterium timepoint measurements in HDX-MS experiments were performed in triplicates on the same sample. All data for differential HDX-MS comparisons were acquired on the same day to minimize instrumental variations. All attempts at replication were successful. |
| Randomization | Samples were injected into the LC-MS system according to the measuring time point (i.e. each measuring time point was analyzed to completion before starting a new time point). Samples were injected in a random order within the individual time points. |
| Blinding | Blinding was not relevant for this study. No live subjects were involved and the sample preparations performed in an experimental laboratory. |

# Reporting for specific materials, systems and methods

We require information from authors about some types of materials, experimental systems and methods used in many studies. Here, indicate whether each material, system or method listed is relevant to your study. If you are not sure if a list item applies to your research, read the appropriate section before selecting a response.

### Materials & experimental systems

| n/a | Involved in the study | |
|---|---|---|
| ☒ | ☐ | Antibodies |
| ☒ | ☐ | Eukaryotic cell lines |
| ☒ | ☐ | Palaeontology and archaeology |
| ☒ | ☐ | Animals and other organisms |
| ☒ | ☐ | Human research participants |
| ☒ | ☐ | Clinical data |
| ☒ | ☐ | Dual use research of concern |

### Methods

| n/a | Involved in the study | |
|---|---|---|
| ☒ | ☐ | ChIP-seq |
| ☒ | ☐ | Flow cytometry |
| ☒ | ☐ | MRI-based neuroimaging |
